# An ultra-high-stability four-axis ultra-high-vacuum sample manipulator

**DOI:** 10.1107/S1600577521004859

**Published:** 2021-06-08

**Authors:** Marcus Agåker, Carl-Johan Englund, Peter Sjöblom, Nial Wassdahl, Pierre Fredriksson, Conny Såthe

**Affiliations:** a Physics and Astronomy, Uppsala University, PO Box 516, SE-75120 Uppsala, Sweden; b MAX IV Laboratory, Lund University, PO Box 118, SE-22100 Lund, Sweden; c Englund Engineering AB, Kättinge 25, 755 92 Uppsala, Sweden

**Keywords:** beamline, manipulator, high precision, ultra-high vacuum

## Abstract

A report on a four-axis ultra-high-stability manipulator developed for use at the Veritas and Species RIXS beamlines at MAX IV Laboratory, Lund, Sweden, is presented. The manipulator consists of a compact, light-weight *X–Y* table with a stiffened *Z* tower carrying a platform with a rotary seal to which a manipulator rod holding the sample can be attached. Its design parameters have been optimized to achieve high eigen-frequencies via a light-weight yet stiff construction, to absorb forces without deformations, provide a low center of gravity, and have a compact footprint without compromising access to the manipulator rod. The manipulator system can house a multitude of different, easily exchangeable, manipulator rods that can be tailor-made for specific experimental requirements without having to rebuild the entire sample positioning system. It is shown that the manipulator has its lowest eigen-frequency at 48.5 Hz and that long-term stability is in the few tens of nanometres. Position accuracy is shown to be better than 100 nm. Angular accuracy is in the 500 nrad range with a long-term stability of a few hundred nanoradians.

## Abstract 2

An ultra-high-stability four-axis manipulator for use with soft X-ray beamlines, exhibiting eigen-frequencies in the 50 Hz region and a linear/angular positional accuracy below 100 nm/500 nrad, is described. Long-term positional stabilities of 20 nm/200 nrad are also demonstrated.

## 1. Introduction   

Veritas and Species are two resonant inelastic X-ray scattering (RIXS) beamlines at MAX IV Laboratory, Lund, Sweden, that operate in the energy range 30–1500 eV (Såthe *et al.*, 2020 ▸; Urpelainen *et al.*, 2017 ▸; Sjöblom *et al.*, 2020 ▸). RIXS is a technique used in a broad range of applications (Schmitt *et al.*, 2014 ▸; Rubensson, 2015 ▸; Rubensson *et al.*, 2013 ▸; Nordgren & Rubensson, 2013 ▸) but in recent years the use of RIXS in the study of correlated materials has led to an expansive build up of high-resolution soft X-ray RIXS beamlines world wide (Schmitt *et al.*, 2013 ▸; Brookes *et al.*, 2018 ▸; Diamond Light Source, 2020 ▸; Jarrige *et al.*, 2018 ▸; Dvorak *et al.*, 2016 ▸), where the main focus has been the study of solid samples. At MAX IV, the stated goal of the RIXS beamlines has been to offer as wide sample capabilities as possible to cater to a broad community of users.

The new storage ring designs at MAX IV (Tavares *et al.*, 2014 ▸) have made it possible to achieve very small foci at the experimental stations. This has eliminated the need for source-defining entrance slits to the instruments that are now operating in ‘slitless mode’. Using the beam spot on the sample directly as source for the spectrometer optics requires a stable beam and a stable sample. Motions affecting the spot position on the sample or the samples position along the beam propagation direction will change the source position and source size for the spectrometer optics, shifting the energy scale and compromising the instruments resolving power. The issue of beam stability has been address by Agåker *et al.* (2020 ▸); here we report on the development of a four-axis ultra-high-stability manipulator to be used with the slitless X-ray spectrometers at MAX IV.

In the soft X-ray range, radiation is rapidly absorbed by any material and so experiments must be conducted in vacuum, thereby setting very specific requirements on sample manipulators. Sample manipulation can be conducted by either external systems moving the sample from outside vacuum or internal systems mounted inside the vacuum vessels. Traditionally sample manipulation at soft X-ray synchrotron end-stations has been performed using external four-axis manipulators (Diamond Light Source, 2020 ▸; Jöhri *et al.*, 2016 ▸) with a fixed or one-/two-axis manipulator rod holding the sample. High-stability sample manipulation has been achieved using in-vacuum goniometers (ESRF, 2020 ▸) or hexapods (PhysicInstrumente, 2020 ▸) with stacked motion tables sharing a common center point where the sample can be mounted. Systems of this design are usually quite bulky, limiting both the sample volume and accessibility. These assemblies are also quite complex and by necessity need to be fully housed inside the experimental chamber. This limits samples to those compatible with ultra-high vacuum (UHV) so as to prevent contamination of movable parts.

Traditional manipulators consisting of stacked *X–Y* tables with a *Z* tower and a top-mounted rotary platform are, from their general design, not very stable constructions. This is in part due to their high center of gravity and limited space to support the top platform symmetrically as well as the long distances from the mounting point to the sample position. The dimensions of the edge-welded bellows, required to allow movement while maintaining UHV, also limit the clear bore of these assemblies and hence the space available for the manipulator rod.

Early investigations into manipulators for the Veritas and Species RIXS experimental stations found that no commercially available solutions presented quantitative measures of stability and precision at the sample point, despite the precision of constituent components seeming at first glance to be very adequate. Therefore, finding a system with a stable sample point combined with the versatility demands set by the rest of the beamline specifications and the spatial restraints set by nearby equipment was difficult to accomplish.

As a result, the decision was made to develop a new manipulator implementing novel engineering solutions and exploiting the experience accumulated by the beamline team and stability research performed at MAX IV Laboratory. This development was carried out in a collaboration between MAX IV, Englund Engineering AB and Uppsala University. The goal was to combine the versatility of an in-air positioning system with the stability of an in-vacuum goniometer or hexapod, whilst simultaneously allowing a wide variety of sample environments to be used but still offer the necessary stability needed for slitless spectrometers.

The chosen design is similar to the commonly used *X–Y–Z* manipulators. It consists of a stacked *X–Y* table base structure, on top of which a *Z*-tower is mounted, supporting a movable platform holding a rotary platform where the sample rod is attached. The design has been optimized to allow the *Z*-platform to rest very close to the *X–Y* base during operation. With this configuration the sample rod and the experimental environment can easily be exchanged by removing the rod from the top of the manipulator, giving the system the required sample flexibility. In the measurement presented in this text a simple sample holder is used but does not mean that the manipulator is limited to this sample environment.

For stability purposes, it was considered imperative to keep weight to a minimum and to locate the center of gravity as close to the manipulator mounting point as possible. High-strength aluminium alloys were cut into a truss design throughout for maximum strength to weight ratio. The design concept chosen allowed unibody properties (unitary construction design) to be achieved, and careful consideration of the functionality of each part eliminated unstable add-on solutions. A three-pillar design was initially considered, and, although it could have been made as rigid as the chosen design due to the full symmetry around the *Z*-axis, it lacked sample accessibility for the same reason. The final unibody design concept, with a back plate for the vertical motion, on the other hand permits free access to the top of the *Z*-platform in a 190° arc, regardless of *X*, *Y* and *Z* positions. This limits the angular stroke to a 190° arc if parts of the sample rod assembly protrude outside the rotation radius of 63 mm. However, if no parts protrude outside the rotation radius there is a 360° rotational freedom of movement.

**Figure 1:**
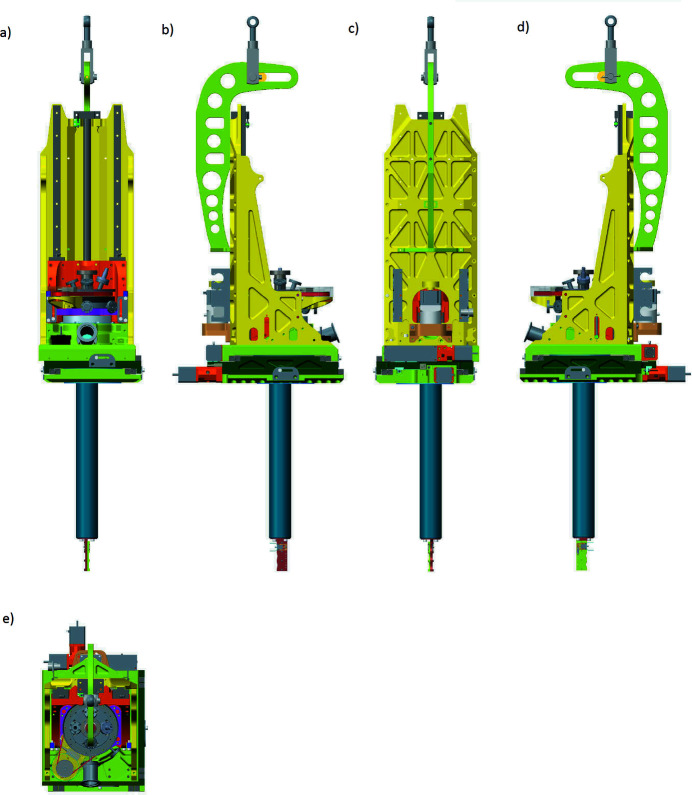
(*a*) Front view, (*b*) left side, (*c*) rear view, (*d*) right side and (*e*) top of the manipulator. The perforated lifting arm seen in panels (*b*) and (*d*) at the top of the manipulator can be removed as it is not part of the operation of the manipulator.

All motors and external equipment have been tucked in close to the center of the manipulator, whose relatively small volumetric size and low weight also simplify handling and usage. The manipulator footprint as seen from its top is only 300 mm × 260 mm. An overview of the manipulator is shown in Fig. 1 ▸ showing the manipulator from the front, sides, top and rear. The total build height is 740 mm, excluding lift arm [perforated L-shape seen in Fig. 1 ▸, panels (*b*) and (*d*)].

Another feature that was highly prioritized was simple sample handling and exchange. To this extent, the system was fitted with an easy detachable sample rod system that facilitates pre-preparation of sample environments and systems. Should the manipulator itself need to be removed, an integrated balance-block can be adjusted on the lifting arm so that it always hangs in a true vertical position.

## 2. Layout   

**Figure 2:**
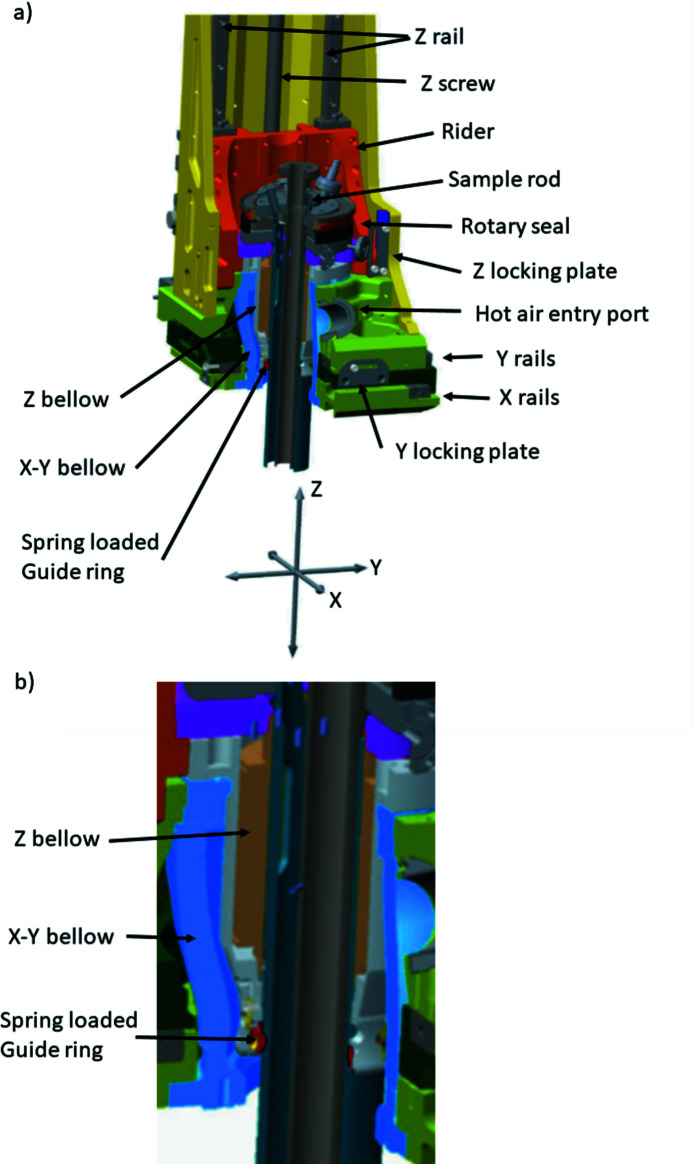
(*a*) Cutaway of the internal structure of the manipulator showing the rider with the rotary seal, the optional lock-plates, edge-welded bellows, spring loaded (floating) support guide ring (marked in red) and the manipulator rod. The KF40 hot air entry port for baking out the bellows is also shown. (*b*) Close-up of the bellows arrangement showing the *Z*-bellows (brown) inside the *X–Y* bellows (blue). The spring-loaded guide ring is shown in red.

Fig. 2 ▸ depicts the cross section of the lower part of the manipulator in the intended working positions. Some key components of the design such as the *X–Y* table and rider together with the two edge welded bellows and the spring-loaded guide ring are marked in the graphics.

### 2.1. *X–Y* table   

The *X–Y* table consists of three aluminium plates where each plate is separated from the next by cross roller guides (see Fig. 2 ▸). The *X–Y* motion is driven by stepper motors (Oriental, 2020 ▸) attached to precision ball screws mounted inside and on the side of the plates, respectively. In the center, a clear bore allows a 127/157 mm (inner/outer diameter) edge-welded bellows from ComVAT to be mounted, allowing full, simultaneous, ±15 mm strokes in the *X* and *Y* directions, respectively (see Fig. 2 ▸). A Renishaw absolute encoder (Renishaw, 2020 ▸) monitors the motion. Electrical switches from Pulsotronic are used as travel limiters. The mounting face of the *X–Y* base has provisions for extra support plates for mounting the manipulator, besides the CF100 flange connection. These supports are custom made to fit to the chosen experimental chamber and increase the stability of the system. An integrated KF entry port sits on the top of the *X–Y* table for heated air bake out of the bellows, see Fig. 2 ▸. Hot air leak-out ports help establish controlled, uniform, heat build-up.

### 2.2. *Z*-tower   

The *Z*-tower consists of a back plate machined out of a solid aluminium plate. The back plate is stabilized by two triangular side plates, connecting the back plate and the *X–Y* base [see Fig. 1 ▸, panels (*b*), (*c*) and (*d*)]. The back plate holds two linear motion rail guides (THK, 2020*a* ▸), each consisting of a rail with two blocks. A rider platform, see Fig. 2 ▸, is attached to the four blocks, holding the rotary seal and the base of the sample rod. A precision ball screw from THK (THK, 2020*b* ▸) is mounted between the rails which actuates the *Z*-motion. The working *Z*-stroke is 400 mm with an extra 10 mm at each end as safety margin.

To allow the rider platform, which represents a large part of the mass of the *Z*-tower, to rest as close to the *X–Y* stage as possible during measurements, the compressed *Z*-bellows are partially housed inside the *X–Y* bellows. This moves the center of gravity closer to the intersection between the base plate and the *Z*-tower than what could be done with a single bellow or traditional stacked bellows arrangement. The separation of the *Z*-motion bellows from the *X–Y* motion bellows also helps reduce the vacuum forces acting on the raider and back plate by the use of a smaller-diameter bellows for the *Z*-motion than what is required for the *X–Y* motion. The stepper motor driving the *Z*-motion is connected to the drive screw via a cog belt drive system. An electric brake is attached between the motor and the cog belt system to prevent unintentional motion in case of power failure. Electrical switches from Pulsotronic are used as travel limiters.

### 2.3. Rotary platform   

A differentially pumped CF 63 rotary platform (Thermionics, 2020 ▸) is attached to the rider of the *Z*-tower, see Fig. 2 ▸. A Nanotec stepper motor (Nanotec, 2020*a* ▸) with attached gearbox (Nanotec, 2020*b* ▸) engages a cog belt drive that rotates the platform. Cog belts are used in this manipulator whenever an off-axis drive is needed for their low-maintenance needs and cleanliness. A Renishaw absolute angular encoder measures the angle.

### 2.4. Sample rod   

**Figure 3:**
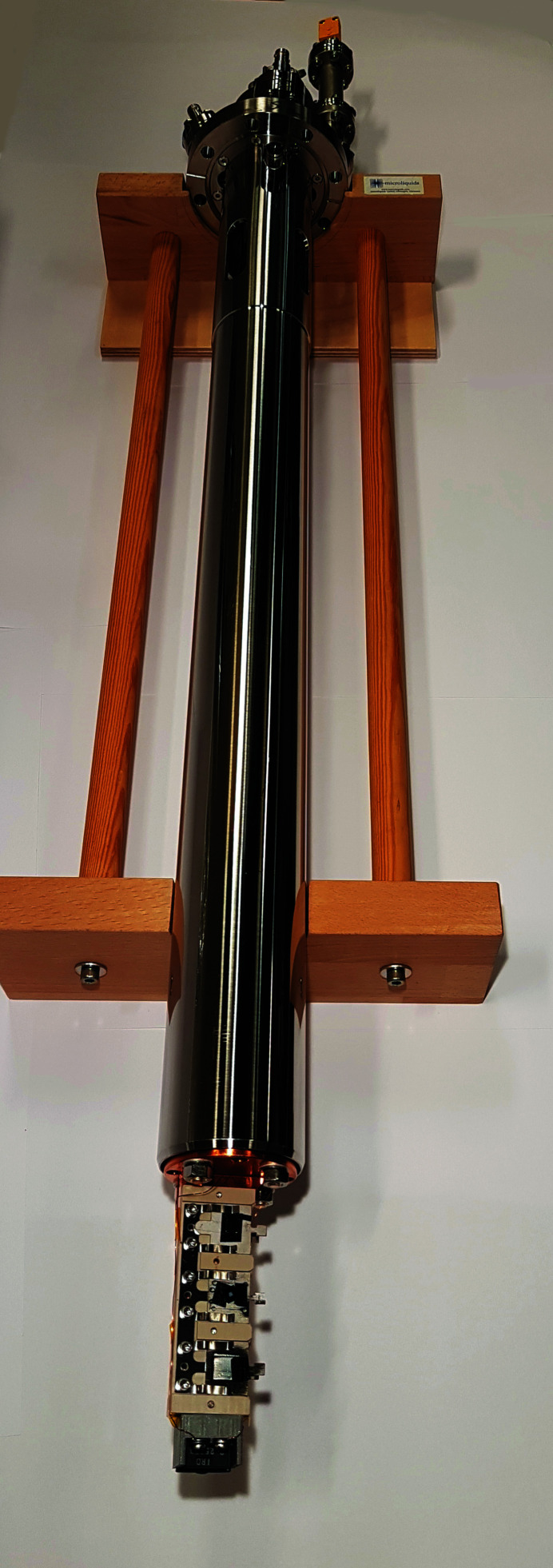
Sample rod used in the measurements presented in this text. It consists of an outer guide tube and an inner tube for LN (empty in the measurements). The sample holder consists of a copper rod that is attached to the LN reservoir. It has room for three samples that are in contact with the copper and three samples that sit on an isolated surface [polyetheretherketone (PEEK)].

The CF 63 flange of the rotary platform acts as the main attachment point for the sample manipulator rod holding the sample and/or the sample environment. This sample rod was intentionally kept simple, yet sturdy, and is exchangeable to enable a wide variety of sample environments. The sample rod nominal diameter is 58 mm which is the maximum diameter that could be used within a CF 100 flange while still allowing the desired ±15 mm *X–Y* stroke. Thin wall tubing was chosen to minimize the weight of the rod. The rod interacts with a spring-loaded floating PTFE (polytetrafluoroethylene) guide ring, acting as a secondary support at the bottom of the *Z*-tower bellows (see Fig. 2 ▸). This secondary support point reduces the distance from the sample position to the attachment point, and also serves to stiffen the rod base by adding some extra leverage. This increased stiffness also makes the rod resilient to lateral forces during sample transfers, using flag-type sample plates and spring-loaded retaining clamps. The system can still be used with smaller-diameter rods but with the loss of the secondary attachment point unless a matching diameter guide ring is used. The guide ring is easily detachable and can be modified/replaced to suit other sample rod dimensions. The length of the rod is 602 mm, optimized to allow the *Z*-tower to be fully compressed at its lowest position. This corresponds to the measurement position inside the Veritas experimental chamber (Englund *et al.*, 2015 ▸). For Species RIXS, a slightly shorter rod can be used due to a shorter distance between manipulator attachment and beam spot. For simplicity, the same manipulator rods are used for both beamlines, allowing easy sharing of sample environments. The rod used in the following measurements consists of the outer guide tube and an inner tube, used for liquid-nitrogen (LN) cooling (empty for the measurements presented here). The sample holder consists of a copper plate attached to the bottom of the LN reservoir, see Fig. 3 ▸. Apart from this rod there is currently (2021) also a rod with a fixed sample position closed-cycle He cryostat from ColdEdge (Coldedge, 2020 ▸), a rod with a liquid beam and catcher from Microliquids (Microliquids, 2020 ▸), a gas-/liquid-flow cell from Uppsala University. A two-axis version of the He cryo-rod is under development, making the system (full) six-axis, as well as a He-buffered gas beam and a cell for electrochemistry. Other sample environments can be adopted for the system as needed. To further increase stability during measurements, solid brass brackets can be temporarily mounted between the rider platform and the base of the manipulator (see Fig. 2 ▸), locking it in place and enabling a more symmetric support of the rider platform whilst minimizing the *Z*-bellows vacuum force effect of bending the back plate. The working position can be adjusted by changing the brass rods to the appropriate length (Veritas and Species have different working positions). The slits in the locking plates allow a 50 mm stroke without changing the brass rods. The *X* and *Y* axes also have similar locking plates that are designed to work over the whole *X–Y* range. None of the stability measurements presented in the subsequent sections utilized these options though. The locks can also be used to secure and protect the drive actuators during transportation and handling.

## 3. Stability   

As part of the stability work at MAX IV, a stability policy has been implemented stating that all components influencing resolution, spot size and beam position shall not have any natural eigen-frequencies below 55 Hz. This is to prevent any of the naturally occurring background vibrations at the lab exciting eigen-modes of the equipment, and thereby compromising the performance of the beamlines as a whole. During the construction phase of the MAX IV facility, a green-field survey (Rothschild, 2009 ▸; Agåker *et al.*, 2020 ▸) of the Brunnshög site in north-eastern Lund was performed by the Norwegian Geological Institute. It was concluded that the amplitude of most of the ground vibrations were of the order of 40–60 nm and occurred at 5–20 Hz. Setting the limit at 55 Hz gives a safety margin both for the occurrence of natural vibrations at higher frequencies and for the mechanical systems ability to conform to the requested eigen-mode frequencies.

### 3.1. Resonances   

**Figure 4:**
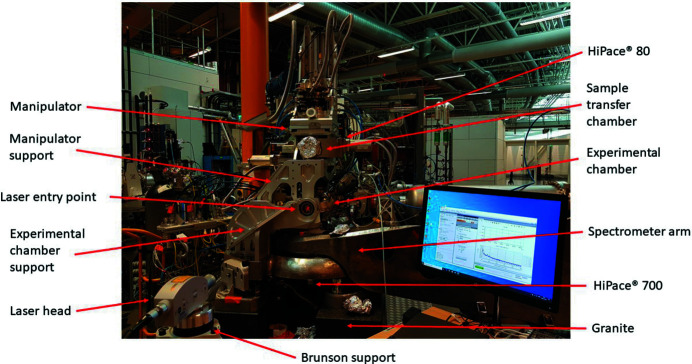
The vibrational measurement setup is shown as well as the manipulator mounted on the Veritas beamline. Note the laser dot reaching the sample holder through the glass window. The beamline stretches upstream in the background and the spectrometer arm stretches out to the right.

To characterize the resonances present in the manipulator a Polytec laser interferometer (Polytec, 2020 ▸) was used to measure the naturally occurring vibrations in the system as well as the eigen-modes. The interferometer was also used to track the motion of the system during controlled translations. The laser head is a 1 mW cw^−1^ laser with λ = 633 nm mounted on either a short or long tripod support (Brunson). The laser head was connected to an OFV 543 laser controller which in turn was connected to a Polytec OFV-500 vibrometer controller. The analog output signal from the vibrometer was fed to a Data Translation DT 9837A hardware, controlled by *QuickDAQ* software. Measurements were performed with the manipulator under vacuum, 5 × 10^−9^ mbar, and attached to the Veritas endstation. A Pfeiffer High Pace 80 turbo­molecular pump was attached in direct connection to the manipulator through the sample transfer chamber situated directly under the manipulator. A High Pace 700 turbomolecular pump was attached to the experimental chamber, separated from the manipulator by an edge-welded bellow. The experimental chamber and the manipulator are supported on separated stands with a common base. Their contribution cannot be seen directly in the measurements but might be present indirectly by exciting the manipulator eigen-modes. The measurement setup is shown in Fig. 4 ▸ together with the manipulator mounted on top of the Veritas experimental chamber (Englund *et al.*, 2015 ▸). The granite support, which is under-grouted to the floor, creates the support structure for both the manipulator and spectrometer arm. The experimental station is situated in the main experimental hall at MAX IV, and is subject to the ambient environment.

**Figure 5:**
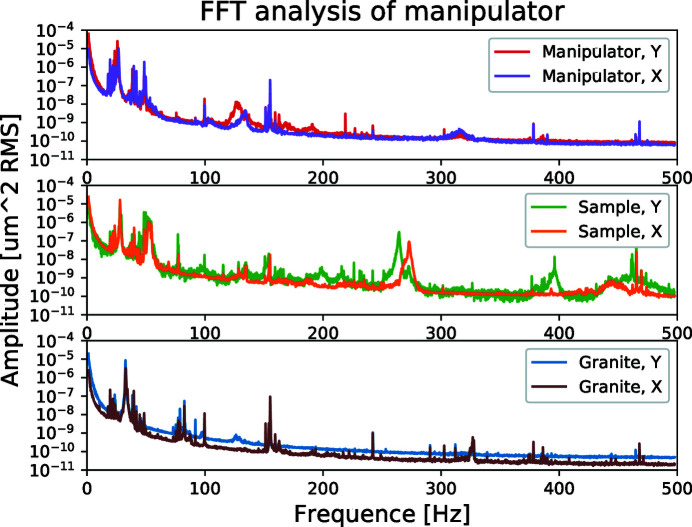
Measured fast Fourier transform (FFT) spectra on the manipulator granite, frame and sample holder in vacuum through glass windows.

Vibration measurements were performed on the granite base of the endstation, the manipulator and the sample, see Fig. 5 ▸. Note that the laser beam going to the sample holder, where it is directly reflected of the steel springs holding the sample plates, goes through the glass windows on the chamber. The interferometer supports used have their own eigen-modes that shows up in the measured spectra and has to be accounted for in the analysis. The measurements for the granite were made with a shorter Brunson (model 231), while the manipulator and sample were measured with a longer model (model 230), with slightly variable length. The short Brunson model had eigen-modes around 33 Hz while the long one had modes at 28 Hz or 26 Hz, respectively, depending on length. This explains why the 33 Hz peak seen in Fig. 5 ▸ (bottom panel) is shifted to 28 Hz for the sample measurement (middle panel) and 26 Hz for the manipulator (top panel).

Measurements on the granite, Fig. 5 ▸, show a strong contribution at 33 Hz followed by a number of resonances at approximately 38.5, 39.8, 42.0, 44.8, 46, 48.5, and 49.9 Hz with smaller amplitude for higher frequencies. In this measurement, no vibrations are intentionally induced but instead reflect the naturally occurring background vibrations at the lab.

**Figure 6:**
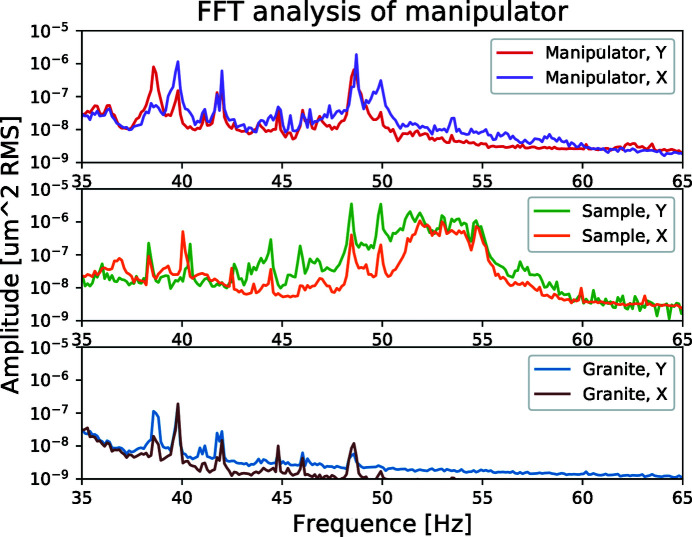
Same measured FFT spectra as in Fig. 5 ▸, but for 35–65 Hz instead of 0–500 Hz for visibility. At the sample, the first important frequency is 48.5 Hz. The 48.5 Hz and 49.9 Hz frequencies originate from the manipulator while the 51–55 Hz cluster originates from the rod.

Close inspection of the granite and manipulator measurements (Fig. 6 ▸) show that the 38.5 Hz and 39.8 Hz resonances do not shift with the length of the Brunson and have therefore a different origin. As they are the dominant resonances in the granite measurement they are the granite support eigen-frequencies in the *X* and *Y* axes, respectively. The resonances are also visible in the sample measurement, but with 10 times lower amplitude and 30 times lower than the dominant vibrations present in the sample, *i.e.* very suppressed and not contributing to the performance.

In the manipulator measurements, 48.5 and 49.9 Hz resonances are present, with the 49.9 Hz being more dominant in *X* than in *Y*. Both resonances are also present in the sample measurement but it seems that the vibrations in *X* are suppressed as compared with *Y* which could be explained by the stiffer walls in this direction as a result of the manipulator structure. In the sample measurements, there is a cluster of resonances ranging from 51 to 55 Hz that are not present elsewhere in the system and are therefore likely to be originating in the rod itself. They are equally strong in the *X* and *Y* directions as the rod is (more or less) symmetrical.

Summarizing the vibration measurements, the lowest frequency to give a contribution at the sample is 48.5 Hz while the resonances ranging from 51 to 55 Hz will be dependent on the rod in use.

Resonance measurements have only been conducted in the *X–Y*-plane as there were no means to reliably deflect the laser in the vertical direction.

## 4. Motion   

The laser interferometer used in the vibration measurements was also used to externally verify that the linear motion of the sample is the same as the motion seen by the absolute encoders located on the frame of the manipulator and that it is free of overshoot and ringing. The interferometer is focused on the sample holder inside the vacuum system, recording its motion at the same time that the control system makes defined positioning moves.

### 4.1. Linear motions   

**Figure 7:**
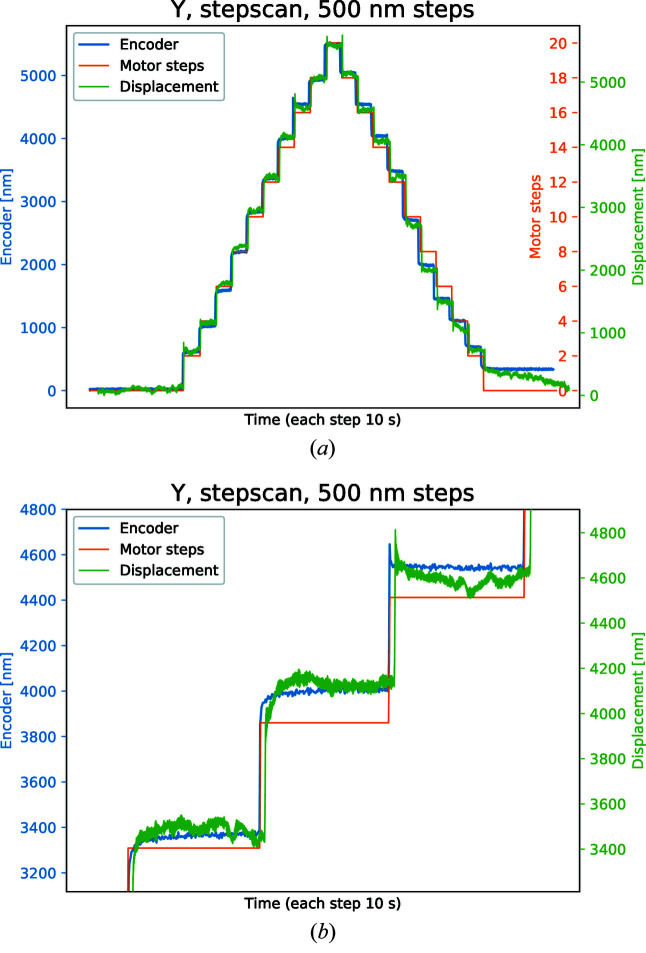
(*a*) Step scan in the *Y* direction where a laser interferometer verifies the motion by focusing on the sample holder and compares it with the absolute encoder located on the frame of the manipulator. (*b*) Same motion as in (*a*) but zoomed in on three steps. When the encoder shows a smooth approach to target position, the interferometer does the same. When the encoder shows a spike, the interferometer does the same.

In Fig. 7 ▸, the *X* motion is carried out in 500 nm steps monitored both by the laser interferometer as well as the absolute position encoder. Each 500 nm movement corresponds to two motor steps with a deadband of one motor step, *i.e.* 50% of the 500 nm step is within tolerance and no compensation motion will occur. In later measurements, the level of micro-stepping is increased to reduce drifting and provide a higher level of accuracy. Nevertheless, the external measurement overlaps very well with the absolute encoder, which is easier to verify in the zoomed figure, Fig. 7 ▸(*b*). When the encoder shows a smooth approach to target position, the interferometer does the same. When the encoder shows a spike, the interferometer does the same. Examining the first step in Fig. 7 ▸(*b*), the full width at half-maximum (FWHM) of the interferometer is 57 nm while the absolute encoder shows a 19 nm FWHM. The interferometer shows a larger FWHM partially because the measurements are taking place at the end of the rod, at the sample position, rather than at the base, where the absolute encoder is located, but also because the interferometer measurement setup is very sensitive to, for example, variation in ambient temperature from ventilation and people. The FWHM measurement suggests that the smallest meaningful step for the motor to take is about 50 nm, and would therefore set the resolution limit.

**Figure 8:**
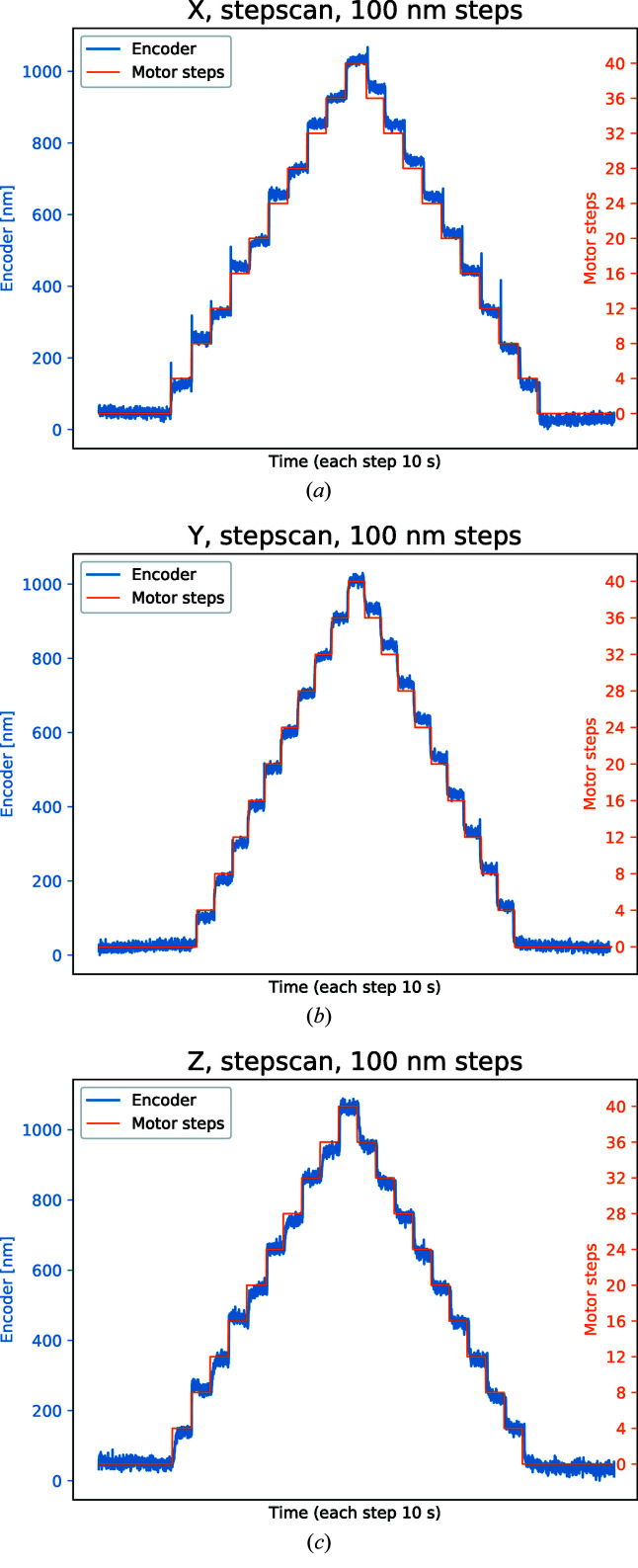
Staircase motion with 100 nm steps in the (*a*) *X*, (*b*) *Y* and (*c*) *Z* direction. The noise level is only a fraction of the step, and the original position is reached after the motion.

To approach the limit, the micro-stepping of the motors was increased to 25 nm steps and thus the smallest deadband. A set of staircase motion measurements are made for the *X*, *Y*, and *Z* directions as shown in Fig. 8 ▸ where each step is 100 nm, *i.e.* four motor step motions. The interferometer in our setup is not able to track this motion mainly due to temperature fluctuation in the ambient air which causes the measured position to drift back and forth, and is therefore omitted. However, the encoder clearly resolves a 100 nm motion without the noise in one position overlapping with the next position.

### 4.2. Rotary motion   

**Figure 9:**
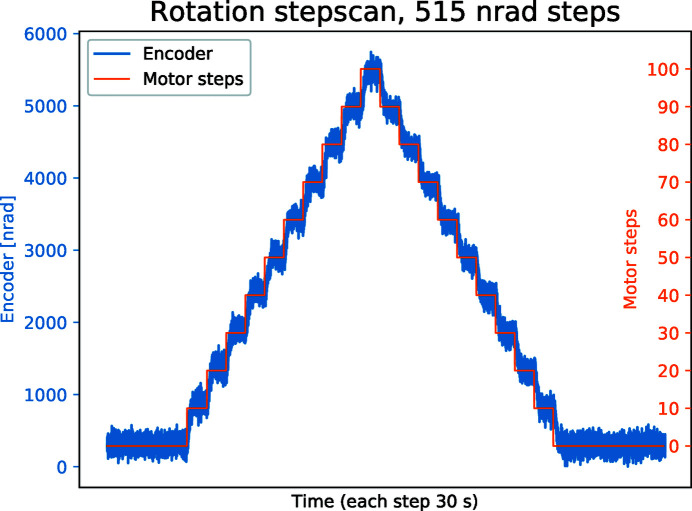
Step scan with 515 nrad steps around the vertical axis, *y* axis (yaw). Each step is 30 s long. The blue line is the absolute encoder while the orange is the individual motor steps.

Not all motions could be monitored with the interferometer and for these the absolute encoders are trusted. In Fig. 9 ▸, the rotation of the sample rod is monitored by its 32 bit (approximately 1.5 nrad per tick) absolute rotary encoder (blue curve), as it rotates in 515 nrad steps around its vertical *Z*-axis. The rotary precision is a byproduct of the mechanical drive train needed to overcome the friction for the rotary seal, and was not set as a specific requirement for Veritas and Species. Each step is 30 s long and it takes about 10 s to settle at the new position. The difference in average positions moving upwards and downwards ranges from 50 nrad to 82 nrad. The difference in average position before and after the up and down motion of the entire staircase is only 12 nrad.

**Figure 10:**
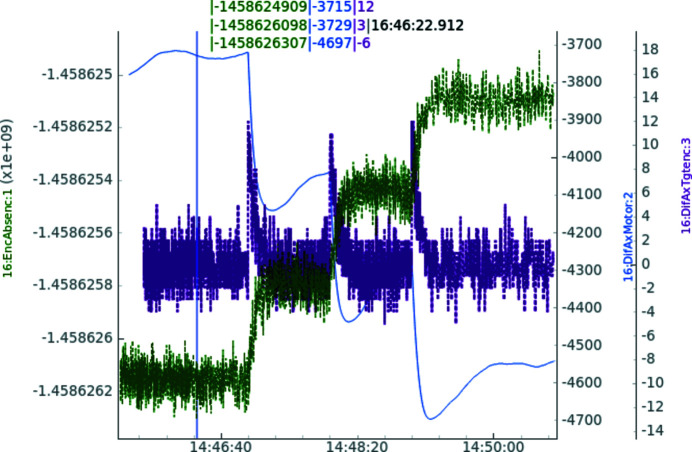
Three steps of 515 nrad rotational motion separated by one minute. The green curve is the absolute encoder position and thus should make three distinct steps. Between motions, the encoder position should remain as still (flat) as possible. The purple curve is the difference between ideal absolute encoder position and its real value and should be minimized by the closed loop. The blue thin curve is the amount of motor steps that the closed loop is forced to introduce to keep the encoder position.

For each 515 nrad rotation motion, the motor takes ten steps but, since it is also under closed-loop control, it is also adjusting its position according to the encoder. The motor is only allowed to differ by one step — corresponding to 50 nrad — from its ideal position as monitored by the encoder, forcing it to a near constant, but slow, motion, as it needs to compensate the drag and slack from the cog belt drive. This behavior is illustrated in Fig. 10 ▸, which also shows how one of MAX IV’s tools for motion control and tuning (Sjöblom *et al.*, 2019 ▸) works when operating the IcePAP motion controller (Janvier *et al.*, 2013 ▸). When the system receives a motion command, a difference between where the encoder is supposed to be according to its projected trajectory and where it actually is appears, which is indicated by the purple curve showing three ten-step deviations. The motor (blue thin curve) is then moving to reduce the encoder difference (purple curve) in a smooth manner without any overshoot or vibration. As the motion is actuated through a cog belt drive, it also needs to compensate for some elastic stretching and relaxing of the rubber as well as overcome the friction forces in the rotary seal. This is shown as backwards motion on the motor to keep the encoder position after the motion. The actual position, indicated by the green encoder curve, approaches its position in a controlled manner. From a scanning perspective, the allowed difference between the desired position and when the new position is considered reached is set as an individual parameter and thus faster scan speed can be achieved at the expense of reduced position accuracy.

### 4.3. Long time stability   

**Figure 11:**
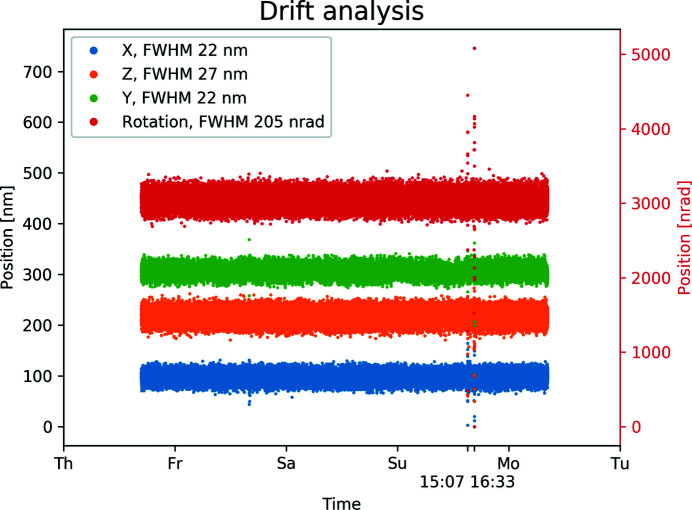
Four days of monitoring the encoders on the manipulator to ensure stability in position. The spike occurring in the Sunday afternoon reveals user interaction with the equipment. No drift is noticed.

A longer, four day, measurement with the aim of understanding the manipulators ability to hold its position over time is shown in Fig. 11 ▸ where the *X*, *Y*, *Z*, and rotation positions are visible as recorded by their respective encoders. The FWHM is 22 nm for the *X–Y* table while for the vertical *Z* direction FWHM it is 27 nm. It is natural that this dimension has slightly larger vibrations due to its upright length and longer distance to the granite support as compared with the *X–Y* table. The rotary encoder shows a FWHM of 205 nrad. The noise is most likely from the encoder environment as the position readout is optical and communication is through data packages, so no electronic interference is expected. No particular hour to hour or day to day cycles were observed.

### 4.4. Spectrometer arm influence during motion   

The granite stone supporting both the weight of the Q-chamber and the manipulator also supports the spectrometer arm. The steel frame constituting the arm is 10 m long, weighs approximately 2 tonnes and can swing 120° to allow scattered photons to be detected in different scattering directions. The spectrometer arm is moved by lifting it from the floor with pressurized air flowing through its lifting feet. A friction-driven tug, moving on a curved steel rail, drags the arm as it is floating on its air cushions to its designated position, where it is lowered to its support feet by carefully reducing the air flow and pressure to zero. It is crucial that the sample is returned to its position after arm movement so as to allow the beam to hit the same spot on the sample.

**Figure 12:**
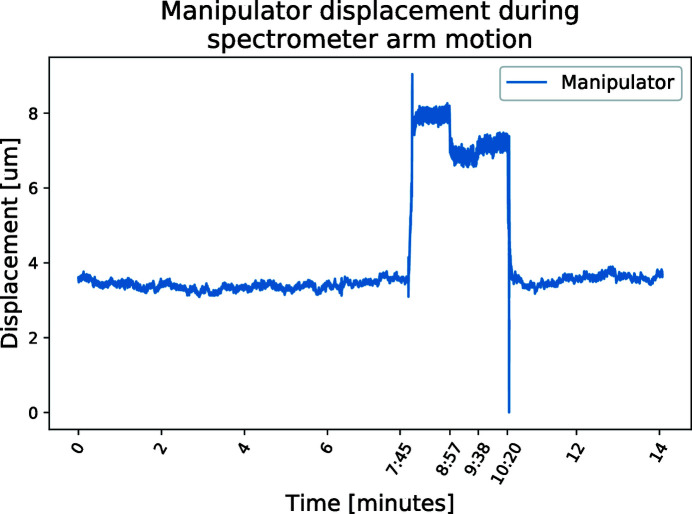
Lifting and moving the spectrometer arm also affects the position of the sample in the manipulator as they share the same support stone. It is crucial that the sample is returned to its position after changing the angle of the spectrometer arm. At 7 min 45 s the spectrometer arm is lifted on its air feet, at 8 min 47 s it is moved 10 cm forward and at 9 min 18 s it is moved the same distance back. At 10 min 20 s the arm was lowered and air flow turned off. In the measurement, the difference in position before and after motion is 180 nm.

In this measurement the interferometer setup is used to monitor the position of the sample. The measurement on the sample, Fig. 12 ▸, is made in the same direction as the spectrometer arm. The measurement starts with a few minutes of resting. Then the arm is lifted and moved 10 cm forward; after a minute of rest it is moved back to the original position and lowered to its support feet. Note that the manipulator has no ringing after the spectrometer arm is returned to its resting position. The sample position is returning to its original position with a difference of 180 nm, calculated as the difference in the average position before and after motion. The noise in the sample position during lifted motion of the arm is larger, but not alarmingly large — 0.5 µm. There is a 3 µm spike indicating the landing of the arm. Note that the noise in this measurement is larger than the noise in the stepping measurements, reflecting the larger dynamic range used for this measurement. The difference is thus most likely due to electronic noise in the readout.

## 5. Hardware overview   

To provide an overview of the manipulator, key parameters are summarized in Table 1 ▸ showing the present status. A total of four such manipulators have — as of 2020 — been built and are always subject to customization and improvement. Therefore, the data in the table cannot be considered to be static.

## 6. Conclusion   

This paper shows that it is possible to build an ultra-high-stability four-axis UHV sample manipulator with the positioning performance of a goniometer, but with significantly increased flexibility in terms of sample holder exchange and vertical motion. The design avoids piezo stages and thus their range limitations and issues with long-term reliability. It also steers clear of unnecessary complexity. Instead, careful planning and optimizing of the selected components, together with thorough identification of the critical design parameters, have been performed.

## Figures and Tables

**Table 1:** Hardware overview

	*X*	*Y*	*Z*	Rotation
Range (mm)	±15	±15	400	360°†
Resolution (nm)	100	100	100	515 nrad
FWHM (nm)	22	22	27	205 nrad
Eigenfrequency (Hz)	48.5	48.5	–	–
Motor‡	PKP246	PKP246	PKP296	ST4209M1704-A
Lead screws§	BNK1002	BNK1002	DIK2004	–
Gears¶	–	–	2:1 cogbelt	GPLE40-3S-256 + 3:1 cogbelt
Brake††	–	–	MAB23x	–
Bearing‡‡	VR6 300	VR6 250	SHS20LV	RNN250
Encoder§§	RL32BAT001	RL32BAT001	RL32BAT001	RA32BAA150

† Unlimited, but in reality limited by the tubes exiting on the top, typically 190°.

‡ Motors from Oriental and Nanotec.

§ All are THK brand.

¶ Cog drives are custom from Aratron, rotation gearbox is Nanotec.

†† JVL brand, only the *Z*-axis has been equipped with parking break due to its weight and vacuum force to prevent freefall in case of power out-take.

‡‡ All are THK brand, except rotation, which is Thermionics.

§§ All are absolute optical Renishaw encoders.
